# A systems approach to evaluate nitrogen utilization efficiency and environmental impacts of swine growing-finishing feeding programs in U.S. pork production systems

**DOI:** 10.1093/jas/skad188

**Published:** 2023-06-06

**Authors:** Zhaohui Yang, Pedro E Urriola, Lee J Johnston, Gerald C Shurson

**Affiliations:** Department of Animal Science, University of Minnesota, St. Paul, MN 55018, USA; Department of Animal Science, University of Minnesota, St. Paul, MN 55018, USA; Department of Animal Science, University of Minnesota, St. Paul, MN 55018, USA; West Central Research and Outreach Center, University of Minnesota, Morris, MN 56267, USA; Department of Animal Science, University of Minnesota, St. Paul, MN 55018, USA

**Keywords:** carcass characteristics, feeding programs, growth performance, life cycle assessment, sustainability, swine

## Abstract

Traditionally, swine diets have been formulated to meet nutrient requirements at the lowest cost with little regard toward minimizing environmental impacts. The overall objective of this study was to evaluate the relative differences among four grower-finisher feeding programs, using precision diet formulation practices, on growth performance, carcass composition, nitrogen utilization efficiency, and environmental impacts. In experiment 1, four 4-phase growing-finishing feeding programs consisting of diets containing corn and soybean meal (**CSBM**), low protein CSBM supplemented with crystalline amino acids (**LP**), CSBM with 30% distillers dried grains with solubles (**DDGS**), and DDGS supplemented with crystalline Ile, Val, and Trp (**DDGS + IVT**) were fed to 288 mixed sex pigs (initial body weight [BW] = 36.9 ± 4.2 kg) for 12 wk to determine effects on growth performance and carcass characteristics. Pigs fed with CSBM had greater (*P* < 0.05) final BW than those fed with LP and DDGS, and greater gain efficiency than pigs fed with LP. Pigs fed with DDGS + IVT tended to have greater (*P* = 0.06) backfat depth than pigs fed with DDGS, and less (*P* < 0.05) loin muscle area than pigs fed with CSBM. In experiment 2, nitrogen (**N**) and phosphorus (**P**) balance of barrows (*n* = 32; initial BW = 59.9 ± 5.1 kg) fed with each of the phase-2 diets from experiment 1 was determined in a 12-d metabolism study (7 d adaptation and 5 d collection). Pigs fed with CSBM had a greater (*P* < 0.05) amount of N retained than pigs fed with other diets, but also had a greater (*P* < 0.05) amount of urinary N excretion and blood urea N than pigs fed with LP and DDGS + IVT diets. Pigs fed with LP tended (*P* = 0.07) to have the greatest N utilization efficiency but the least (*P* < 0.05) P retained as a percentage of P intake among dietary treatments. Diet composition and data collected from experiments 1 and 2 were used to calculate life cycle assessment environmental impacts using Opteinics software (BASF, Lampertheim, Germany). The CSBM feeding program had the least impact on climate change, marine and freshwater eutrophication, and fossil resource use. The LP feeding program had the least impact on acidification, terrestrial eutrophication, and water use, while the DDGS feeding programs had the least impact on land use. These results indicate that feeding CSBM diets optimized growth performance and carcass composition while simultaneously reducing impacts on climate change, marine and freshwater eutrophication, and fossil resource use compared with the other feeding programs evaluated.

## Teaser

Although none of the feeding programs evaluated in this study were superior in every productivity and environmental impact measure, the corn-soybean meal (CSBM) feeding program optimized growth performance of growing-finishing pigs while simultaneously reducing environmental impacts on climate change, eutrophication potential, and fossil resource use compared with CSBM diets containing lower protein content or corn dried distillers grains with solubles.

## Introduction

Long-term sustainability of pork production is dependent on improving profitability, caloric and nutritional efficiency, and the percentage of carcass lean while reducing the environmental footprint. Major advancements in the development and use of accurate feed formulation approaches and precision feeding technologies can greatly enhance profitability, efficiency of energy and nutrient utilization to achieve sustainable production of high quality and safe pork while minimizing negative environmental impacts (Pomar and Remus, 2019; Shurson et al., 2021). Precision feeding of pigs has been shown to reduce feed costs by more than 8%, nitrogen (N) and phosphorus (P) excretion by about 40%, and greenhouse gas emissions by 6% (Andretta et al. 2014, 2016, 2018). However, the global livestock industry contributes about one-third of human-induced N emissions (i.e., nitrates, ammonia, nitrous oxide, and other nitrogen oxides), of which poultry and pork supply chains contribute 29% of total N emissions from food producing animals, and 68% of these N emissions are associated with feed production (Uwizeye et al., 2020). Furthermore, only 10% to 44% of N and 34% of P in feed consumed by pigs is utilized to produce lean pork for human consumption (Gerber et al., 2014).

Nitrogen emissions from pork production systems have several detrimental environmental consequences. Nitrous oxide is a potent greenhouse gas, while ammonia and nitrogen oxides contribute to air pollution, cause acidification and eutrophication, and pose risks to human health (Galloway et al., 2008; Sutton et al., 2013). Nitrates and organic N have caused increased water pollution and biodiversity loss (Erisman et al., 2013; Ascott et al., 2017; Hamilton et al., 2018). Because protein is the second largest cost (after energy) component of swine diets, and about 75% of feed consumed in pork production systems is consumed in the grower-finisher phase, the low efficiency of converting dietary protein into carcass lean and the high environmental impact of various types of N losses require an extensive evaluation of dietary protein feeding strategies for growing-finishing pigs to assess their relative effectiveness for improving the environmental sustainability of pork production.

Soybean meal (SBM) continues to be an excellent dietary protein source for swine because of its complementary amino acid profile with corn, and high amino acid concentrations and digestibility (85% to 94%) compared with other plant-based protein sources (NRC, 2012; Ruiz et al., 2020). Corn distillers dried grains with solubles (DDGS) is produced in large quantities comparable to that of SBM in the United States, and because it is often less expensive than corn and SBM, it is commonly added at levels up to 30% in growing-finishing swine diets as a partial replacement of corn and SBM (Shurson, 2017). However, a recent meta-analysis of 24 published studies (Jang et al., 2021) showed that growing-finishing pigs fed with corn DDGS diets averaged 1.68% less average daily gain (ADG) and 1.06% less gain:feed (G:F) compared with pigs fed with corn-SBM diets. These researchers also found that for each percentage unit increase in dietary DDGS inclusion rate, ADG was reduced by 0.10% and average daily feed intake (ADFI) was reduced by 0.09%. These reductions in ADFI and ADG can be attributed to inefficiencies of N utilization associated with excess Leu relative to Ile and Val (Cemin et al., 2019; Kwon et al. 2019, 2020), excess Leu and Met on Trp utilization (Salyer et al., 2013; Gonçalves et al., 2015; Kwon et al., 2022), and increased endogenous losses of Thr resulting from high fiber concentrations in DDGS (Mathai et al., 2016; Wellington et al., 2018). Therefore, diet formulation adjustments are needed to improve growth performance, carcass lean, and N utilization efficiency when feeding diets containing high amounts (e.g., 30%) of DDGS to growing-finishing pigs.

Several studies have shown that crude protein (CP) content of swine diets can be reduced significantly if adequate crystalline amino acids (CAA) are used to supplement diets formulated on a digestible amino acid basis (Gloaguen et al., 2014; Wang et al., 2018). High protein diets contain excesses of some indispensable AA above the pig’s requirements, which results in excretion of excess N in urine and feces as well as reduced gut health due to protein fermentation in the hindgut (Wang et al., 2018). Studies have shown that dietary CP can be reduced by up to four-percentage units when supplementing diets with L-Lys, DL-Met, L-Thr, and L-Trp, which increases N utilization efficiency, reduces feed cost and nitrogen excretion, and promotes gut health without compromising growth performance of pigs (Kerr et al., 2003; Nyachoti et al., 2006; Yue and Qiao, 2008; Fan et al., 2017). However, feeding low CP diets to growing-finishing pigs can result in increased carcass fat content, which may be a result of insufficient amounts of some AA relative to energy consumed (Kerr et al., 2003). Furthermore, depending on the frequency of meals, studies have shown that digestion and absorption of AA occur more rapidly in diets containing high amounts of CAA than in diets comprised primarily of intact protein sources such as SBM (Yen et al., 2004). Therefore, depending on the amount of SBM replaced with corn and CAA, the efficiency of N utilization may be compromised.

Life cycle assessment (**LCA**) of environmental impacts of food production systems has become a widely accepted reference method for guiding decisions and transitioning toward more globally sustainable food production and consumption patterns (LEAP, 2015). Because of the significant contributions of swine feed to the overall environmental impacts of pork production systems, multi-objective feed formulation is an emerging approach that uses LCA data for feed ingredients to formulate least-cost, nutritionally adequate, low environmental impact swine diets (Mackenzie et al., 2016; Garcia-Launay et al., 2018; Méda et al., 2021; de Quelen et al., 2021). However, limited studies have been conducted to evaluate LCA impacts of feeding programs in U.S. pork production systems. Inconsistent results have been reported from studies evaluating environmental impacts of feeding corn DDGS diets (Lammers et al., 2010; Stone et al., 2012; Haque and Liu, 2019; Benavides et al., 2020), and there is limited information on the environmental impacts from feeding reduced CP diets supplemented with synthetic AA (Lammers et al., 2010; Benavides et al., 2020). Therefore, the overall objective of this study was to evaluate the relative differences among four grower-finisher feeding programs, using precision diet formulation practices, on growth performance, carcass composition, nitrogen utilization efficiency, and environmental impacts.

## Materials and Methods

The experimental protocol used in both animal studies was approved by the University of Minnesota Institutional Animal Care and Use Committee (#2104-38981A).

### Animal and housing

Two experiments were conducted to evaluate growth performance and carcass characteristics (experiment 1), and N and P balance (experiment 2). The first experiment was conducted in an environmentally controlled growing-finishing facility at the University of Minnesota West Central Research and Outreach Center (Morris, MN, USA). Pigs (*n* = 288; initial body weight [BW] = 36.9 ± 4.2 kg) were blocked by initial BW and sex and assigned to pens to provide 9 pigs per pen. Pens within each block were randomly allotted to one of four dietary treatments (feeding programs) to provide 8 pens per treatment. The sex ratio between gilts and barrows within pens was the same for all treatments. Each pen was equipped with two nipple waterers, a four-space self-feeder, and comprised of total-slatted, concrete floors. All pigs had ad libitum access to feed and water throughout the 84-d experimental period. Pigs were observed daily to evaluate health and to ensure that the waterers and feeders were properly functioning.

The second experiment was conducted in an environmentally controlled metabolism facility at the University of Minnesota Southern Research and Outreach Center (Waseca, MN, USA). Growing barrows (*n* = 32; initial BW = 59.9 ± 5.1 kg) were housed individually in metabolism crates with a fully slatted plastic floor, a nipple waterer, and a stainless-steel feeder in each crate. Pigs were randomly assigned to one of four phase 2 diets from the same feeding program treatments used in experiment 1. Pigs were provided an amount of their respective experimental diets equivalent to three times the energy requirement for maintenance (i.e., 197 kcal/kg × BW^0.60^; NRC, 2012) in two equal meals twice daily (0800 and 1600 hours). Water was provided ad libitum through nipple drinkers. Pigs were observed daily to evaluate health and to ensure that pigs consumed all feed offered in each meal and that no wastage occurred.

### Dietary treatments

Corn (locally sourced in Morris, MN, USA), SBM (AG Processing, Dawson, MN, USA), and DDGS (DENCO II, Morris, MN, USA) were each obtained in a single lot and sampled for nutrient and mycotoxin analysis before the experiments were conducted (Table 1). Diets were formulated for a four-phase growing-finishing program (Tables 2 and 3), and diet phase changes were made when the average pig BW of each pen was within 2.5 kg of the target BW of the beginning of the next phase (phase 1 = 25 to 50 kg, phase 2 = 50 to 75 kg, phase 3 = 75 to 100 kg, and phase 4 = 100 to 130 kg). Nutrient requirements for growing-finishing pigs were estimated from the NRC (2012) model using growth performance data from the previous study conducted at the same facility (Hilbrands et al., 2021) as inputs, and adjusting the predicted feed intake for the genetic source used in experiment 1 as recommended by the genetic supplier.

**Table 1.** Chemical composition of major ingredients used in experimental diets (as-fed basis)

Measure	Corn	SBM	DDGS
Dry matter, %	86.0	89.2	85.4
CP, %	7.2	46.4	28.1
Crude fat, %	1.8	0.0	6.1
Neutral detergent fiber, %	6.9	8.6	28.4
Acid detergent fiber, %	2.4	6.6	12.5
Ash, %	1.2	6.4	4.5
Starch, %	70.5	5.2	6.9
Calcium, %	0.005	0.46	0.02
Phosphorus, %	0.26	0.60	0.81
NE^1^, kcal/kg	2,685	2,141	2,132
AA			
Lysine, %	0.23	3.04	1.02
Threonine, %	0.22	1.76	1.05
Methionine, %	0.12	0.61	0.54
Cystine, %	0.14	0.66	0.58
Tryptophan, %	0.07	0.87	0.26
Isoleucine, %	0.25	2.25	1.10
Valine, %	0.32	2.29	1.43
Arginine, %	0.29	3.38	1.22
Histidine, %	0.19	1.23	0.74
Leucine, %	0.77	3.57	3.08
Phenylalanine, %	0.32	2.40	1.41
Tyrosine, %	0.17	1.65	1.00
Mycotoxins^2^			
Deoxynivalenol, ppm	ND^3^	ND	0.4

^1^NE of corn and soybean meal were calculated from chemical composition using equations from NRC (2012); NE of DDGS was calculated from chemical composition using an equation from Wu et al. (2016a).

^2^Corn, soybean meal, and DDGS samples were analyzed for multiple mycotoxins but only deoxynivalenol was detected in DDGS.

^3^ND,not detectable.

**Table 2.** Diet composition and calculated nutrient analysis of phases 1 and 2 diets (as-fed basis)

	Phase 1 diets^1^ (25–50 kg)				Phase 2 diets (50–75 kg)			
Ingredients, %	CSBM	LP	DDGS	DDGS +IVT	CSBM	LP	DDGS	DDGS +IVT
Corn	61.13	74.50	54.07	53.81	68.58	81.34	60.68	60.44
SBM	34.96	22.00	10.58	10.58	27.84	15.50	4.19	4.19
Corn DDGS	–	–	30.00	30.00	–	–	30.00	30.00
CDO	1.40	–	2.00	2.00	1.38	–	2.08	2.08
Monocalcium phosphate	0.48	0.62	0.02	0.02	0.37	0.50	–	–
Calcium carbonate	1.30	1.40	1.81	1.81	1.15	1.24	1.61	1.61
Salt	0.40	0.40	0.40	0.40	0.40	0.40	0.40	0.40
Phytase^2^	0.025	0.025	0.025	0.025	0.025	0.025	0.025	0.025
VTM premix^3^	0.20	0.20	0.20	0.20	0.20	0.20	0.20	0.20
L-Lysine HCl	–	0.42	0.56	0.56	–	0.40	0.54	0.54
DL-Methionine	0.07	0.19	0.11	0.11	0.03	0.15	0.06	0.06
L-Threonine	0.03	0.20	0.18	0.18	0.03	0.20	0.18	0.18
L-Tryptophan	–	–	0.04	0.07	–	–	0.04	0.08
L-Isoleucine	–	–	–	0.06	–	–	–	0.06
L-Valine	–	0.05	–	0.17	–	0.05	–	0.14
TOTAL	100.0	100.0	100.0	100.0	100.0	100.0	100.0	100.0
Calculated analysis								
NE, kcal/kg	2,500	2,500	2,500	2,500	2,544	2,544	2,544	2,544
CP, %	20.7	16.3	18.0	18.2	17.9	13.7	15.5	15.6
ADF^4^, %	3.8	3.2	5.8	5.8	3.5	3.0	5.5	5.5
NDF^5^, %	7.2	7.0	13.2	13.2	7.1	6.9	13.0	13.0
SID AA								
Lys, %	1.05	1.05	1.05	1.05	0.87	0.87	0.87	0.87
Met + Cys, %	0.59	0.59	0.59	0.59	0.49	0.49	0.49	0.49
Thr, %	0.66	0.66	0.66	0.66	0.57	0.57	0.57	0.57
Trp, %	0.30	0.21	0.21	0.24	0.25	0.17	0.17	0.20
Leu, %	1.51	1.19	1.47	1.47	1.33	1.03	1.31	1.31
Ile, %	0.82	0.59	0.61	0.66	0.69	0.47	0.49	0.55
Val, %	0.86	0.68	0.68	0.84	0.73	0.57	0.57	0.70
SID Thr:Lys, %	63	63	63	63	65	65	65	65
SID Trp:Lys, %	29	20	20	23	29	19	19	23
SID Leu:Lys, %	144	113	140	140	153	119	151	151
SID Ile:Lys, %	78	56	58	63	80	54	57	63
SID Val:Lys, %	82	65	65	80	84	66	66	81
Total Ca, %	0.74	0.74	0.74	0.74	0.64	0.64	0.64	0.64
STTD P, %	0.33	0.33	0.33	0.33	0.30	0.30	0.31	0.31

^1^DDGS = CSBM diet with 30% distillers dried grains with solubles; DDGS + IVT = DDGS diet supplemented with L-Ile, L-Val, and L-Trp.

^2^Phytase 2000 (VitaPlus) provided 250 FTU/kg of complete feed with a release value of 0.09% STTD phosphorus.

^3^VTM = vitamin-trace mineral premix; Provided the following amounts per kg of premix: 2,646,000 IU vitamin A; 485,000 IU vitamin D; 13,230 IU vitamin E; 1,380 mg vitamin K; 11 mg vitamin B_12_; 11,000 mg niacin; 8,270 mg pantothenic acid; 3,530 mg riboflavin; 50 g zinc; 50 g iron; 12 g manganese; 6 g copper; 0.2 g iodine; and 0.1 g selenium.

^4^ADF, acid detergent fiber.

^5^NDF,neutral detergent fiber.

**Table 3.** Diet composition and calculated nutrient analysis of phases 3 and 4 diets (as-fed basis)

	Phase 3 diets^1^ (75–100 kg)				Phase 4 diets (100–130 kg)			
Ingredients, %	CSBM	LP	DDGS	DDGS +IVT	CSBM	LP	DDGS	DDGS +IVT
Corn	73.73	85.15	65.14	64.96	78.70	90.32	65.03	65.01
SBM	23.08	12.00	–	–	18.33	7.00	–	–
Corn DDGS	–	–	30.00	30.00	–	–	30.00	30.00
CDO	1.20	–	2.00	2.00	1.20	–	2.57	2.57
Monocalcium phosphate	0.24	0.36	–	–	0.12	0.24	–	–
Calcium carbonate	1.08	1.17	1.47	1.47	1.01	1.10	1.29	1.29
Salt	0.40	0.40	0.40	0.40	0.40	0.40	0.40	0.40
Phytase^2^	0.025	0.025	0.025	0.025	0.025	0.025	0.025	0.025
VTM premix^3^	0.20	0.20	0.20	0.20	0.20	0.20	0.20	0.20
L-Lysine HCl	–	0.36	0.52	0.52	–	0.36	0.37	0.37
DL-Methionine	0.02	0.12	0.04	0.04	–	0.10	–	–
L-Threonine	0.03	0.18	0.16	0.16	0.02	0.17	0.09	0.09
L-Tryptophan	–	–	0.05	0.08	–	0.02	0.03	0.05
L-Isoleucine	–	–	–	0.05	–	0.02	–	–
L-Valine	–	0.03	–	0.10	–	0.05	–	–
TOTAL	100.0	100.0	100.0	100.0	100.0	100.0	100.0	100.0
Calculated composition								
NE, kcal/kg	2,566	2,566	2,566	2,566	2,597	2,597	2,597	2,597
CP, %	16.0	12.3	13.8	13.9	14.2	10.4	13.6	13.6
ADF^4^, %	3.3	2.8	5.3	5.3	3.1	2.6	5.3	5.3
NDF^5^, %	7.0	6.9	13.0	13.0	7.0	6.8	13.0	13.0
SID AA								
Lys, %	0.75	0.75	0.75	0.75	0.63	0.63	0.63	0.63
Met + Cys, %	0.43	0.43	0.43	0.43	0.37	0.37	0.39	0.39
Thr, %	0.50	0.50	0.50	0.50	0.43	0.43	0.43	0.43
Trp, %	0.22	0.14	0.14	0.17	0.19	0.12	0.12	0.14
Leu, %	1.22	0.95	1.21	1.21	1.10	0.83	1.21	1.21
Ile, %	0.61	0.41	0.42	0.47	0.52	0.34	0.42	0.42
Val, %	0.65	0.49	0.50	0.59	0.57	0.42	0.50	0.50
SID Thr:Lys, %	67	67	67	66	68	68	68	68
SID Trp:Lys, %	29	19	19	23	30	19	19	23
SID Leu:Lys, %	163	126	161	161	175	131	192	192
SID Ile:Lys, %	81	55	56	62	83	54	66	66
SID Val:Lys, %	87	65	66	79	91	67	79	79
Total Ca, %	0.57	0.57	0.57	0.57	0.50	0.50	0.50	0.50
STTD P, %	0.26	0.26	0.31	0.31	0.23	0.23	0.31	0.31

^1^DDGS = CSBM diet with 30% distillers dried grains with solubles; DDGS + IVT = DDGS diet supplemented with L-Ile, L-Val, and L-Trp.

^2^Phytase 2000 (VitaPlus) provided 250 FTU/kg of complete feed with a release value of 0.09% STTD phosphorus.

^3^VTM,vitamin-trace mineral premix; Provided the following amounts per kg of premix: 2,646,000 IU vitamin A; 485,000 IU vitamin D; 13,230 IU vitamin E; 1,380 mg vitamin K; 11 mg vitamin B_12_; 11,000 mg niacin; 8,270 mg pantothenic acid; 3,530 mg riboflavin; 50 g zinc; 50 g iron; 12 g manganese; 6 g copper; 0.2 g iodine; and 0.1 g selenium.

^4^ADF,acid detergent fiber.

^5^NDF, neutral detergent fiber.

Experimental feeding programs consisted of 1) corn-soybean meal control diets (**CSBM**), 2) low CP CSBM diets (**LP**) with supplemental CAA to partially replace SBM while meeting all digestible amino acid requirements, 3) CSBM diets containing 30% DDGS (**DDGS**), and 4) 30% DDGS diets supplemented with crystalline L-Val, L-Ile, and L-Trp (**DDGS + IVT**). All diets were formulated on net energy (**NE**), standardized ileal digestible (**SID**) amino acid, and standardized total tract digestible (**STTD**) P basis to meet or exceeded energy and nutrient requirements for growing-finishing pigs. Diets within each phase contained equal NE and SID Lys. Precision diet formulation was based on using dynamically determined NE, SID amino acid and nutrient loading values for corn, SBM and DDGS being fed, and updating certain amino acid requirements based on the most recent publications. Values for NE in corn and SBM were calculated from analyzed chemical composition using published equations (NRC, 2012), and NE for DDGS was calculated using an equation from a previous study (Wu et al., 2016a). NE of corn distillers oil (**CDO**) was assumed to be the same as NE of corn oil in NRC (2012) after comparing reported values in several studies (Kerr et al., 2016; Kellner and Patience, 2017; Lindblom et al., 2017). An anti-oxidant (Rendox CQ, Kemin, Des Moines, Iowa, USA) was added at 0.1% inclusion to the oil tank at the feed mill to prevent lipid oxidation of CDO used throughout the experiment. The amino acid SID co-efficients for DDGS were calculated using prediction equations from (Zeng et al., 2017), while SID co-efficients for corn and soybean meal, and all STTD phosphorus for these ingredients were obtained from NRC (2012). Dietary supplementation levels of L-Val, L-Ile, and L-Trp for DDGS + IVT were determined based on data reported by Kwon et al. (2021). The same SID Thr:Lys ratios were used for diets within each phase and met recommended levels for diets contained high concentrations of dietary fiber (Mathai et al., 2016). Phytase (VitaPlus, Madison, WI, USA) was added to all diets at 250 FTU/kg diet.

### Growth performance and carcass characteristics

For experiment 1, the BW of individual pigs in each pen was recorded at the beginning of the experiment and bi-weekly thereafter until the end of the 84-d experimental period, to calculate ADG. Feed added and removed from feeders was weighed and recorded to calculate feed disappearance as an estimate of ADFI. Pen weight gain and feed disappearance during each weigh period were used to calculate G:F. When average pen BW reached 120 kg, real-time ultrasound measurements (Exago model, Echo Control Medical, Angouleme, France) of backfat (**BF**) depth and longissimus muscle area (**LMA**) between the 10th and 11th ribs were performed by a trained and certified technician on all marketable pigs (*n* = 267). Images were digitized and computed using Biosoft Toolbox II for Swine Software (Version 2.5.0.6; Biotronics Inc., Ames, IA). All pigs were harvested 2 d later at a commercial abattoir (Hormel Foods, Austin, MN). Hot carcass weight (**HCW**) was measured on all pigs immediately after slaughter, and carcass dressing percentage was calculated as (HCW/final BW) × 100. Fat-free lean (**FFL**) percentage of each carcass was calculated using the NPPC (2000) equation for unribbed carcasses including 10th rib LMA and BF derived from real-time ultrasound along with HCW.

### Nitrogen and phosphorus balance

For experiment 2, the total experimental period included a 7 d adaptation period and a 5 d collection period. The pigs were fed only the Phase 2 diets (50 to 75kg BW) used in experiment 1 for the 12 d experimental period, and the diets contained 0.5% titanium dioxide as an indigestible marker to calculate the digestibility of N and P. Screens for fecal collections were placed below the slatted floor and pans for urine collection were placed under the screens for daily collection of samples in each metabolism cage. Fresh fecal samples were collected twice daily and stored in labeled zip lock plastic bags. Urine was collected daily in plastic buckets containing 50 mL of 3 N HCl to prevent loss of volatile compounds. Fecal samples and 5% of the collected total urine volume were stored at −20 °C immediately after collection. Blood samples were collected from the jugular vein of pigs on the last day of the experiment and centrifuged at 1500 × *g* for 15 min at 4 °C to separate the serum, and to determine blood urea nitrogen (**BUN**) and creatinine concentrations, as additional measures of dietary N utilization efficiency. Urea nitrogen and creatinine concentrations in serum samples were quantified in triplicate using commercial colorimetric detection kits (Invitrogen, ThermoFisher Scientific, Frederick, MD, USA). Individual pig BW was recorded at the beginning and at the end of the 12 d experiment along with the amount of daily feed supplied and feed refusals. After the 5 d collection period, fecal samples were dried at 55 °C in a forced air oven for 24 h and ground through a 1 mm screen. Feces and feed samples were analyzed at the University of Missouri Agricultural Experiment Station Chemical Laboratories (Columbia, MO, USA) for analyses including titanium dioxide, dry matter, N, and P, and urine samples were analyzed for N concentration.

The NRC (2012) model was used to calculate the predicted N and P intake, retention, and excretion for growing pigs from 25 to 50 kg BW. The phase 2 diet formulation for each feeding program was entered into the NRC (2012) model to evaluate the feeding programs for growing-finishing pigs. The NRC (2012) feed ingredient library was used, and corn oil was used as a proxy for CDO because no data are provided in NRC (2012) for this ingredient.

### Life cycle assessment

Environmental impacts of pork production systems under each feeding program were determined for a cradle to farm gate LCA analysis using Opteinics software (BASF, ). Systems boundaries included crop cultivation and feed production, on-farm animal raising and manure management, and slaughterhouse processing, as well as associated impacts of transportation between steps. Diet composition, N and P retention, growth performance, and carcass composition data obtained from experiments 1 and 2 were used as inputs in this LCA model. The Opteinics LCA software follows International Organization for Standardization standards (ISO 14040, 2006), the European Union Product Environmental Footprint Category Rules (PEFCR; European Commission, 2017), the FAO Livestock Environmental Assessment and Performance Partnership (LEAP, 2015) guidelines, and uses LCA data for feed ingredients from the Global Feed LCA Institute (GFLI; https://globalfeedlca.org/) and emissions factors from the Intergovernmental Panel on Climate Change (IPCC, 2006). The functional unit was defined as 1,000 kg of carcass weight for all environmental impact outputs. Specific characterization methods followed guidelines from FAO (LEAP, 2015), European Union PEFCR (European Commission, 2017), and Reckman et al. (2013).

For each feeding program, feed ingredients used in the diet formulations and their origin (country and states) were entered as inputs into the Opteinics LCA model. Emissions associated with each ingredient, except for CDO, L-Ile, and SBM, were obtained from the GFLI database. When region-specific data were not available in the GFLI database, country-specific or global average data for an ingredient was used. Corn germ oil was used as a proxy for CDO, and L-Val was used as a proxy for L-Ile. Because the GFLI database does not have LCA data for soybean meal produced in the United States, environmental impacts of soybean meal were estimated by BASF using internal methods and state-level soybean production data (see Supplementary Data).

Geographic origin of feed ingredients, feed mill location (MN, USA), and farm location (MN, USA) were used as inputs to calculate the emissions associated with the transportation components of the Opteinics model. Transportation of feed ingredients included truck, train, and ship according to their origins and transportation distances. Transportation of pigs to nearby slaughter plants was assumed to have an average transportation distance of 100 km. Estimates of environmental impacts associated with transportation were obtained from the Gabi Database (Sphera Solutions GmbH, Leinfelden-Echterdinge, Germany).

Because the objective of this study was to compare the LCA impacts of growing-finishing swine feeding programs, related factors such as feed mill, housing system, and manure management system were kept constant using default values in Opteinics. The model default values assumed that the feed mill uses 0.0039 kWh/kg feed of electricity, the pigs come from sow farms that produce 28 piglets/sow/yr, and the pig farm uses 14.11 kWh of electricity, 0.96 L diesel, 1.4 L liquefied petroleum gas, 15.62 L oil, 6.5 L drinking water, 1.37 L cooling water, and 0.8 L cleaning water to produce each pig. Pig manure was assumed to be stored in anaerobic deep pits for more than 1 mo and eventually applied to agricultural land to improve soil fertility.

Nitrogen emissions from manure during housing and storage were calculated from the amount of N excreted and volatile solids produced using methods from IPCC (2006) and Reckmann et al. (2013), and the amount of P excreted in manure associated with water eutrophication was estimated according to Reckmann et al. (2013). In general, a tier 2 approach was used for the calculations in manure management, and energy use was incorporated into the overall energy demand of the farm. Depending on soil nutrient concentrations, the application of animal manure to soils can either be used as a “residue” (amendment) to increase nutrient concentrations in nutrient deficit agricultural soils, or as a “waste” that causes nutrient overburden of crop land. The Opteinics model considers manure to be a waste by default, but we considered both scenarios where emissions were allocated to the crop system when manure was considered a “residue”, and emissions were allocated to the pork production system when manure was considered a “waste”.

Our LCA analysis include several environmental impact categories. These measures included climate change, ozone depletion, acidification, terrestrial eutrophication, marine eutrophication, freshwater eutrophication, particulate matter, water use, fossils resource use, and land use, which are consistent with environmental measures of feed ingredients from the GFLI database.

### Statistical analysis

Normality of data generated in experiments 1 and 2, and presence of outliers were evaluated using UNIVARIATE procedure of SAS (SAS Inst. Inc., Cary, NC). Growth performance and carcass data were analyzed in a randomized complete block design using the GLIMMIX procedure of SAS. The model included the fixed effect of diet, and the random effect of block, with pen serving as the experimental unit. Repeated measurement was used to determine effects of feeding program on growth performance data collected over time. For carcass characteristics, HCW was used as a covariate for BF, LMA, and FFL%. Nitrogen and phosphorus balance and blood data were analyzed in a completely randomized design using the GLIMMIX procedure of SAS, and individual pigs were considered as the experimental unit. Dietary treatments were considered as fixed effects, and pigs were considered as random effects. All means are reported as least square means with a Tukey adjustment. Results were considered significant at *P* ≤ 0.05 and trends were reported when 0.05 < *P* ≤ 0.10.

## Results

### Growth performance

During the 12 wk growth performance experiment, the west central region of Minnesota where the study was conducted experienced the worst heat wave on record during June 3 to11, 2021, according to the Minnesota Department of Natural Resources. Average hourly temperature in the barn for that week was 30.1 °C, and average daily maximum temperature was 36.3 °C. Although proper ventilation and cooling sprinklers were used, all pigs were subjected to heat stress during this 1 wk time period, which uniformly reduced feed consumption and growth rate compared with optimal environmental conditions. However, there was no effect of feeding program on ADG or ADFI over the 12 wk feeding period, but pigs fed with CSBM and DDGS + IVT diets had greater G:F than pigs fed LP diets (*P* < 0.01; Table 4). There was a trend (*P* = 0.08) for a feeding program effect on pig BW, where differences in BW among treatments began on day 56 of the feeding period and continued until the end of the experiment, which resulted in pigs fed with CSBM having greater final BW than pigs fed with LP and DDGS diets (P < 0.05). At the end of the experiment, pigs fed with CSBM (123.6 kg) were 2.6 kg heavier than pigs fed with DDGS (121.0 kg), and 2.1 kg heavier than pigs fed with LP (121.5 kg). Pigs fed with DDGS + IVT (123.4 kg) were 2.4 kg heavier than pigs fed with DDGS (121.0 kg) without supplemental L-Val, L-Ile, and L-Trp, although there were no statistical differences in feed intake.

**Table 4.** Effects of four-phase feeding programs^1^ on growth performance of growing-finishing pigs

Measure	CSBM	LP	DDGS	DDGS + IVT	SEM
BW, kg					
Initial	37.1	37.0	36.7	36.7	0.8
Day 14	49.8	49.1	49.9	48.9	0.8
Day 28	63.1	62.6	62.6	62.3	0.8
Day 42	79.7	78.4	78.3	78.6	0.8
Day 56	96.1^x^	94.6^x,y^	94.0^y^	94.7^x,y^	0.8
Day 70	108.9^a^	107.6^a,b^	106.7^b^	107.4^a,b^	0.8
Day 84	123.6^a^	121.5^b,c^	121.0^c^	123.4^a,b^	0.8
*P*-value					
Treatment	0.08				
Time	<0.01				
Treatment × Time	0.22				
Average daily gain, kg/d					
Days 0–14	0.91	0.88	0.95	0.87	0.05
Days 14–28	0.96	0.95	0.91	0.95	0.05
Days 28–42	1.17	1.13	1.12	1.15	0.05
Days 42–56	1.17	1.16	1.12	1.15	0.05
Days 56–70	0.93	0.93	0.91	0.94	0.05
Days 70–84	1.04	0.99	1.01	1.05	0.05
Overall	1.03	1.01	1.00	1.02	0.01
*P*-value					
Treatment	0.17				
Time	<0.01				
Treatment × Time	0.95				
Average daily feed intake, kg/d					
Days 0–14	1.74	1.82	1.83	1.75	0.07
Days 14–28	2.21	2.30	2.21	2.16	0.07
Days 28–42	2.57^ab^	2.65^a^	2.44^b^	2.59^ab^	0.07
Days 42–56	2.76	2.80	2.75	2.73	0.07
Days 56–70	2.89	2.95	2.88	2.96	0.07
Days 70–84	2.88	2.88	2.91	2.95	0.07
Overall	2.51	2.57	2.50	2.53	0.04
*P*-value					
Treatment	0.22				
Time	<0.01				
Treatment × Time	0.22				
Gain:Feed					
Days 0–14	0.52	0.48	0.52	0.50	0.02
Days 14–28	0.44	0.42	0.42	0.45	0.02
Days 28–42	0.46	0.42	0.46	0.44	0.02
Days 42–56	0.43	0.42	0.41	0.42	0.02
Days 56–70	0.32	0.32	0.32	0.32	0.02
Days 70–84	0.36	0.34	0.35	0.36	0.02
Overall	0.42^a^	0.40^b^	0.41^a,b^	0.41^a^	0.01
*P*-value					
Treatment	<0.01				
Time	<0.01				
Treatment × Time	0.89				

^1^DDGS = CSBM diets with 30% distillers dried grains with solubles; DDGS + IVT = DDGS diets supplemented with L-Ile, L-Val, and L-Trp.

^a,b,c^Values with uncommon superscripts within rows differ (*P *< 0.05).

^x,y^Values with uncommon superscripts within rows tend to be different (*P* < 0.10).

### Carcass characteristics

There were no differences in HCW, carcass yield, or carcass FFL percentage among the four feeding programs (Table 5). Pigs fed with DDGS + IVT tended (*P* = 0.06) to have greater BF thickness than pigs fed with DDGS, and pigs fed with CSBM had greater (*P* < 0.05) LMA than those fed with DDGS + IVT.

**Table 5.** Effects of four-phase feeding programs^1^ on carcass characteristics of growing-finishing pigs

Item	CSBM	LP	DDGS	DDGS +IVT	SEM	*P*-value
HCW^2^, kg	94.9	93.5	92.4	94.8	1.9	0.14
Dressing percent, %	76.8	77.0	76.4	76.9	0.6	0.77
BF depth, mm	19.96^x,y^	19.86^x,y^	18.66^y^	20.28^x^	0.61	0.06
Loin muscle area, cm^2^	46.35^a^	45.23^a,b^	44.33^a,b^	44.29^b^	0.74	0.03
Carcass FFL percentage, %	51.48	51.39	51.61	50.85	0.43	0.34

^1^DDGS = CSBM diets with 30% distillers dried grains with solubles; DDGS + IVT = DDGS diets supplemented with L-Ile, L-Val, and L-Trp.

^2^HCW was used as a covariant for BF depth, loin muscle area, and FFL percentage.

^a,b,c^Values with uncommon superscripts within rows differ (*P *< 0.05).

^x,y^Values with uncommon superscripts within rows tend to be different (*P* < 0.10).

### Nitrogen and phosphorus balance

No differences in feed intake were observed among dietary treatments (Table 6). Pigs fed with CSBM had greater (*P* < 0.05) N intake/d, N retained/d, and urinary N excreted/d than pigs fed with LP, DDGS, and DDGS + IVT. Pigs fed with DDGS and DDGS + IVT had a greater (*P* < 0.05) amount of N excreted in feces than pigs fed CSBM and LP. Pig fed LP also had the lowest (*P* < 0.05) amount of total N excretion among all feeding programs, which was 33% lower than that of pigs fed with CSBM. There was no difference in biological value of N among these diets, but pigs fed with LP tended to have the greatest (*P* = 0.07) N retention rate. No differences were observed for serum creatinine concentrations, but BUN concentrations were less (*P* < 0.01) in pigs fed with LP and DDGS + IVT than in pigs fed with CSBM.

**Table 6.** Comparison of in vivo determined nitrogen and phosphorus intake, retention, and excretion with the calculated retention from NRC (2012) model, and blood urea nitrogen and creatinine of pigs fed with phase 2 grower diets^1^

Items	CSBM	LP	DDGS	DDGS +IVT	SEM	*P-*value
Feed intake, kg/d	2.01	1.93	1.95	1.94	0.06	0.5285
N intake, g/d	54.89^a^	40.35^c^	48.26^b^	49.19^b^	1.39	<0.0001
N in feces, g/d	7.36^b^	6.33^b^	9.48^a^	9.11^a^	0.58	<0.0001
N in urine, g/d	15.93^a^	9.21^b^	12.12^b^	11.96^b^	1.35	0.0004
N excreted, g/d	23.28^a^	15.53^b^	21.59^a^	21.06^a^	1.32	<0.0001
N retained, g/d	31.61^a^	24.81^b^	26.67^b^	28.13^b^	1.27	0.0001
N digestibility, %^2^	86.60^a^	84.25^a,b^	80.50^c^	81.38^b,c^	2.28	0.0002
Net protein utilization, %^3^	57.75	61.44	55.26	57.14	2.76	0.073
Apparent biological value, %^4^	66.75	72.92	68.75	70.23	1.28	0.176
P intake, g/d	8.43^a^	6.79^c^	7.71^b^	7.14^b,c^	0.29	<0.0001
P in feces, g/d	4.39	4.14	3.94	3.83	0.26	0.1614
P retention of intake, %	47.89^a^	39.10^b^	49.08^a^	46.37^a,b^	2.89	0.0079
Predicted N retention^5^, %	30.23	38.12	36.77	36.52	–	–
Predicted P retention^5^, %	32.39	34.25	42.21	42.36	–	–
BUN^6^, mg/dL	10.41^a^	5.90^b^	8.18^a,b^	6.79^b^	1.23	0.0061
Creatinine, mg/dL	1.62	1.59	1.81	1.83	0.25	0.6983

^1^DDGS = CSBM diet with 30% distillers dried grains with solubles; DDGS + IVT = DDGS diet supplemented with L-Ile, L-Val, and L-Trp.

^2^N digestibility = N digested/ N intake = (N intake—N feces)/ N intake × 100.

^3^Net protein utilization = N retained/ N intake = (N intake—N feces—N urine)/ N intake × 100.

^4^Apparent biological value = N retained/ N digested = (N intake—N feces—N urine)/ (N intake—N feces) × 100.

^5^Predicted N and P retention from the NRC (2012) model for a 50–75 kg pig.

^6^BUN, blood urea nitrogen.

^a,b,c^Values with different superscripts within the columns are different (*P *< 0.05).

Pigs fed CSBM had the greatest (*P* < 0.01) P intake/d while pigs fed with LP had the least (*P* < 0.01) P intake/d. However, no differences were observed in fecal P concentrations among dietary treatments. Pigs fed LP had less (*P* < 0.01) P retention rate than pigs fed with CSBM and DDGS.

The NRC (2012) predicted N retention rates of these feeding programs ranged from 30.23% to 38.12%, which were consistently less than the actual N retention rates obtained in experiment 2, which ranged from 55.26% to 61.44%. Similarly, the predicted P retention rates (32.39% to 42.36%) were lower than the actual P retention rates (39.10% to 49.08%) determined in vivo.

### Environmental impacts

The CSBM feeding program resulted in the lowest impact per 1,000 kg of carcass weight on climate change, marine eutrophication, freshwater eutrophication, and fossil resource use (Table 7). Using CSBM as the reference feeding program, the LP feeding program resulted in a decrease in acidification (−10.9%), terrestrial eutrophication (−11.2%), water use (−7.6%), and land use (−9.8%), but increased impacts associated with climate change (+3.3%) and fossil resource use (+3.7%). Both DDGS and DDGS + IVT feeding programs increased impacts on climate change (+18.4% to 18.7%), fossil resource use (+42.7% to 47.3%), and water use (+47.2% to 50.0%), but decreased impacts associated with acidification (−3.0% to 3.4%) and land use (−27.2% to 27.3%) compared with the CSBM feeding program.

**Table 7.** LCA of selected environmental impacts (per 1,000 kg of carcass weight) of pork production systems using different growing-finishing four-phase feeding programs and considering manure produced as waste in the Opteinics model

	Feeding program^1^			
Items	CSBM	LP	DDGS	DDGS + IVT
Climate change, kg CO_2_ equiv.	3,470	3,558	4,083	4,093
Ozone depletion, kg CFC-11 equiv.	0.000022	0.000027	0.000021	0.000028
Acidification, mol H + equiv.	110.0	98.0	106.7	106.2
Terrestrial eutrophication, mol N equiv.	484.5	430.2	465.4	462.0
Marine eutrophication, kg N equiv.	44.32	44.72	45.06	44.72
Freshwater eutrophication, kg P equiv.	23.13	24.18	26.51	26.35
Particulate matter, disease incidence	0.000859	0.000780	0.000838	0.000835
Water use, m^3^ water equiv.	1,678	1,551	2,516	2,468
Fossil resource use, MJ	19,257	19,974	27,475	28,357
Land use, soil quality index	254,870	229,801	185,680	185,291

^1^DDGS = CSBM diets with 30% distillers dried grains with solubles; DDGS + IVT = DDGS diets supplemented with L-Ile, L-Val, and L-Trp.

When considering manure as a “residue” rather than a “waste”, five environmental impact categories, including climate change, acidification, terrestrial eutrophication, marine eutrophication, and particulate matter, had reduced values, while other impact values remained unchanged (Table 8). However, the relative rankings of the four feeding programs in each environmental impact category remained the same regardless of whether manure was considered to be a “residue” or “waste” in the Opteinics model.

**Table 8.** LCA of selected environmental impacts (per 1,000 kg of carcass weight) of pork production systems using different growing-finishing four-phase feeding programs and considering manure produced as residue in the Opteinics model

	Feeding program^1^			
Items	CSBM	LP	DDGS	DDGS + IVT
Climate change, kg CO_2_ equiv.	3,412	3,521	4,036	4,046
Ozone depletion, kg CFC-11 equiv.	0.000022	0.000027	0.000021	0.000028
Acidification, mol H + equiv.	95.4	88.5	94.72	94.25
Terrestrial eutrophication, mol N equiv.	419.5	387.9	411.9	408.5
Marine eutrophication, kg N equiv.	41.16	42.66	42.46	42.12
Freshwater eutrophication, kg P equiv.	23.13	24.18	26.51	26.35
Particulate matter, disease incidence	0.000758	0.000714	0.000755	0.000752
Water use, m^3^ water equiv.	1,678	1,551	2,516	2,468
Fossil resource use, MJ	19,257	19,974	27,475	28,357
Land use, soil quality index	254,870	229,801	185,680	185,291

^1^CAA; DDGS = CSBM diets with 30% DDGS; DDGS + IVT = DDGS diets supplemented with L-Ile, L-Val, and L-Trp.

## Discussion

### Precision diet formulation

It is essential to use the most accurate measures and estimates of the actual nutritional composition of feed ingredients used in diet formulations when implementing precision nutrition strategies to optimize nutrient utilization efficiency in pork production systems (Pomar and Remus, 2019). For example, the NE system is more accurate than the metabolizable energy system because it minimizes overestimation of energy utilization in high-fiber ingredients and in low CP diets (Shurson et al., 2021). Results from recent studies (Cemin et al., 2020; Yang et al., 2020) have indicated that estimates of NE and SID of AA in SBM and DDGS provided by the NRC (2012) may be underestimated. Therefore, NE and SID amino acid values for corn, SBM, and DDGS were dynamically estimated using prediction equations and chemical composition data from analysis for the specific ingredient sources used, rather than static published values from NRC (2012), when formulating diets in this study. Prediction equations to estimate NE concentrations of corn and SBM were based on those proposed by Noblet et al. (1994) as reported in NRC (2012). However, Wu et al. (2016b) reported that the prediction equations developed by Noblet et al. (1994) resulted in suboptimal estimation of NE in DDGS sources, which led to the subsequent development of a more accurate equation (Wu et al., 2016a) that more accurately estimates NE concentrations of DDGS sources. The SID co-efficients for AA in corn and SBM by NRC (2012) were applied to the analyzed AA concentrations of the lots of these ingredients, but because of greater variation in amino acid concentrations and digestibility among DDGS sources, prediction equations from Zeng et al. (2017) were used to estimate SID amino acid content in DDGS used in experimental diets.

### Growth performance

Results from several studies have shown that dietary CP can be reduced by up to four- percentage units, if adequate amounts of L-Lys, DL-Met, L-Thr, and L-Trp are supplemented, to reduce feed cost, N emissions and excretion in manure (Cappelaere et al., 2021), and improve gastrointestinal health (Luise et al., 2021) without compromising growth performance of pigs (Kerr et al., 2003; Nyachoti et al., 2006; Yue and Qiao, 2008; Fan et al., 2017). However, when CP concentrations of LP diets fed in our study were reduced by about four-percentage points in all phases compared with CP concentrations in CSBM diets, final BW of pigs fed with LP was 2.1 kg less and overall G:F was less than for pigs fed with CSBM diets. Conflicting results have been reported on the effects of reducing dietary CP level by more than three-percentage units on growth performance of pigs (Wang et al., 2018). Reduced growth performance in pigs fed with LP diets can be attributed to a lack of intact protein and its hydrolyzed peptides, which is associated with maintaining N retention and whole-body homeostasis (Guay et al., 2006), improving activities of digestive enzymes (Shimizu, 2004), and reducing excessive oxidation of AA (Yen et al., 2004). Depending on the frequency of meals, studies have shown that digestion and absorption of AA occurs more rapidly in diets containing high amounts of CAA than in diets comprised primarily of intact protein sources such as SBM (Yen et al., 2004). In addition, amino acid utilization efficiency may be different in low CP diets if the dietary ideal protein amino acid profiles do not match the changing requirements of pigs during each growth stage (van Milgen and Dourmad, 2015). Therefore, depending on the amount of SBM replaced with corn and CAA, the efficiency of N utilization may be compromised.

The precision formulation approach we used for the 30% DDGS diets was effective in preventing reductions in ADG, ADFI, and G:F, which has been observed in several studies using suboptimal formulation approaches for DDGS diets (Jang et al., 2021). However, final BW of pigs fed with DDGS was 2.6 kg less than those fed with CSBM. Results from a meta-analysis of growth performance data from published DDGS studies showed that for each percentage unit increase in dietary DDGS inclusion rate, ADG is reduced by 0.1% and ADFI is reduced by 0.09% compared with feeding CSBM diets. These results are consistent with results from a recent study by Anderson et al. (2021) that showed a reduction in ADG and G:F in growing pigs when SBM was partially replaced with DDGS and CAA in diets. Similarly, Holen et al. (2021) showed that G:F and final BW were improved as dietary SBM inclusion rate increased in diets containing DDGS and CAA. In both the Anderson et al. (2021) and Holen et al. (2021) studies, the NE system was used in diet formulation and no differences in ADFI was observed, which is consistent with findings in the current study and may suggest that the NE system should be used when formulating diets with DDGS to avoid reductions in ADFI.

Although feeding the 30% DDGS diets resulted in reduced final BW compared with feeding CSBM, our diet formulation approach demonstrates that this reduction in final BW can be overcome by adding L-Val, L-Ile, and L-Trp to the DDGS + IVT diets. One of the challenges of using high dietary inclusion rates of corn DDGS is that corn protein contains excessive Leu relative to Lys and other branched-chain amino acid (Val and Ile) concentrations, which causes an amino acid imbalance that leads to reduced growth performance in pigs (Cemin et al., 2019; Yang et al., 2019). Results from recent studies have shown that supplementing L-Val, L-Ile, and L-Trp to growing finishing diets may partially alleviate the feed intake and growth depression caused by high concentrations of Leu found in diets containing high inclusion rates of DDGS (Cemin et al., 2019; Kwon et al., 2021). Interestingly, Clizer et al. (2021) evaluated the use of SBM to adjust branched-chain amino acid concentrations in diets containing high inclusion rates of corn co-products. Although we did not evaluate this feed formulation approach in the current study, it may be another potential solution to overcome suboptimal growth performance resulting from a dietary BCAA imbalance without the need and cost of supplementing diets with L-Val and L-Ile.

### Carcass characteristics

Feeding low CP diets to growing-finishing pigs can sometimes result in increased carcass BF thickness (Kerr et al., 1995; Ruusunen et al., 2007; Morazán et al., 2015), which may be a result of insufficient amounts of some AA relative to energy consumed (Kerr et al., 2003). However, this effect is not likely to be observed when diets are formulated on a NE basis (Wang et al., 2018). In the current study, all experimental diets were formulated on a NE basis and no differences in carcass BF were observed for pigs fed CSBM and LP feeding programs. It is also possible that a more extreme reduction in dietary CP content may be needed to observe differences in carcass BF than used in the LP feeding program.

Although pigs fed with CSBM and DDGS + IVT had similar final BW, the smaller LMA observed in pigs fed with DDGS + IVT suggests that the gain in BW was a result of increased adipose tissue rather than protein accretion in muscle tissue. This observation is supported by the increased final BW due to the addition of L-Val, L-Ile, and L-Trp to DDGS + IVT diets compared with feeding the DDGS diets without these supplemental CAA, but also resulted in greater carcass BF in pigs fed DDGS + IVT. Therefore, the use of Val, Ile, and Trp may improve BW gain, but may not increase lean muscle deposition. Previous studies (Madeira et al., 2014; Duan et al. 2016, 2018) were conducted to study BCAA supplementation in low protein diets and results showed inconsistent muscle and fat deposition responses. However, all of these studies involved feeding low protein diets in which BCAA could become the next limiting amino acid, and therefore, supplementation resulted in higher protein deposition. In the current study, the supplementation of L-Val, L-Ile, and L-Trp was used to alleviate the negative effects of excessive Leu coming from corn co-products, and therefore, resulted in different outcomes. More studies are needed to better understand and define optimal requirements of branched chain AA in growing-finishing pig diets fed with high amounts of CP from corn co-products.

### Nitrogen and phosphorus balance

One of the solutions to sustainable feeding is adopting precision feeding practices to overcome inefficiencies in nutrient utilization, especially for N and P. Nitrogen and phosphorus intake, retention, and excretion were determined by feeding phase 2 diets representative of each of the four feeding programs. The four-percentage unit reduction in CP concentration in the LP diet compared with the CSBM diet resulted 33% less total N excretion, which is in agreement with results from a previous review which concluded that every percentage unit reduction in dietary CP can reduce N excretion by 7.5% (Wang et al., 2018). Diets containing excess AA greater that the pig’s requirements result in excess N excretion in urine and feces, but using CAA to replace a portion of SBM and reduce CP concentrations minimizes excess N excretion (Wang et al., 2018; Pomar et al., 2021). Pigs fed the LP diet also had about 40% lower BUN levels compared to pigs fed with CSBM, which indicates improved N utilization efficiency of feeding LP diets. As expected, feeding the DDGS and DDGS + IVT diets resulted in lower N digestibility compared with feeding the CSBM diets because of the relatively high CP to Lys ratio, high fiber content, and amino acid imbalance in DDGS compared with SBM (Stein and Shurson, 2009).

No differences were observed in fecal P excretion among Phase 2 grower diets evaluated in this study because all diets contained phytase and were formulated on a STTD P basis. These results indicate that the assumptions used for STTD P concentrations in corn, SBM, and DDGS were accurate, along with the estimated release of P from the phytase source added to these diets. The DDGS and DDGS + IVT diets ­contained no inorganic P (monocalcium phosphate) supplementation. Compared with corn and SBM, DDGS contains much greater concentrations of total and digestible P (NRC, 2012), and adding phytase to swine diets can increase P digestibility by 20% to 50% which subsequently reduces P excretion in manure (Selle and Ravindran, 2008; Humer et al., 2015; Lautrou et al., 2021). Pigs fed with the LP diet had lower P retention than pigs fed the other diets. Although N and P utilization have been generally considered to be independent, results from some studies have shown that P digestion and absorption may be reduced when CP is reduced in swine diets (Xue et al., 2017; Wang et al., 2018). Kebreab et al. (2016) estimated the environmental impacts of feeding swine diets containing CAA and phytase and reported a 35% reduction in eutrophication potential compared with unsupplemented diets, but the contribution from phytase was minimal (3%) based on the assumption that soil P content did not exceed the capacity for crop uptake and the reduction of P in manure would be compensated using inorganic fertilizer. Therefore, because of various assumptions used in calculating estimates of environmental impacts associated with swine diet composition, feeding programs, and subsequent effects on manure composition, caution must be used when attempting to apply those results to real-world production conditions.

The N and P retention rates of the phase 2 grower diets determined in vivo in the current study were compared with predicted N and P retention rates from diet composition using the NRC (2012) model. The N and P retention rates observed in vivo are in agreement with values reported from other studies (Kerr et al., 1995; Otto et al., 2003; Esteves et al., 2021), and substantially greater than the NRC (2012) model predicted values. Therefore, it appears that assumptions used in the NRC (2012) model need to be revised to improve the accuracy of estimation N and P retention rates from diet composition. The NRC (2012) model assumes that pigs require a constant amount of N and P for BW gain regardless of diet composition and feed intake. Therefore, nutrient retention expressed as kg of N or P/kg BW gain is unchanged, but nutrient retention as percentage of intake can vary based on feed intake and feed wastage. This comparison demonstrates the value of accounting for differences in feed intake, growth rate, and lean gain efficiency when evaluating the N and P utilization of various growing-finishing swine diets and feeding programs because accuracy of diet formulation, feeding practices, and environmental conditions on farms affect the actual energy and nutrient requirements, whole-body protein and lipid deposition, and body composition (Dourmad et al., 1998; NRC, 2012; van Milgen and Dourmad, 2015). The differences between observed and predicted N and P retention rates observed for all diets evaluated in this study indicate that the simplified predictions from the NRC (2012) model have limitations and are not representative of responses observed in vivo.

### Environmental impacts

To have the greatest impact on reducing the environmental footprint of pork production, an increased emphasis is needed on feed ingredient sourcing and diet composition because it is the greatest contributor and accounts for 55% to 75% of the climate change impacts, 70% to 90% of energy use, and 85% to 100% of land use attributed to pork production systems (MacLeod et al., 2013; Dourmad et al., 2014; FAO, 2018). Types and sources of feed ingredients and diet formulation practices affect various environmental impacts including global warming, eutrophication, acidification, land occupation, water use, and fossil fuel use among others (Monteiro et al., 2016; van Middelaar et al., 2019; Andretta et al., 2021). Pig productivity and nutrient utilization efficiency of diets and feeding programs can also affect the overall environmental impacts of pork production (McAuliffe et al., 2016). Therefore, a feeding program with the lowest environmental impacts per unit of feed may not result in the least environmental impact per unit of pork produced if growth rate, feed conversion, and percentage of carcass lean are compromised.

LCA is an internationally accepted framework for determining environmental impacts of agricultural production systems (Caffrey and Veal, 2013), and the Global Feed LCA Institute has developed the largest database of LCA data for feed ingredients. However, some LCA data in this database are not representative of ingredients used in U.S. swine diets and are based on country averages that do not account for regional or local differences (Notarnicola et al., 2016). As a result of using these LCA data in the Opteinics model, environmental impact estimates of diets are different than those reported in previous studies evaluating U.S. swine growing-finishing feeding programs (Lammers et al., 2010; Stone et al., 2012; Haque and Liu, 2019; Benavides et al., 2020, Shurson et al., 2022). However, accurate comparisons of LCA data among studies is difficult because of differences in assumptions used in model estimation including system boundaries (e.g., cradle to farm gate or slaughter or retail or human consumption), attribution method (i.e., mass, economic, and energy), allocation of inputs between primary and secondary feed byproducts and co-products (e.g., ethanol vs. DDGS), and manure handling, storage, and application rate relative to nutrient needs in agricultural soils.

In the current study, the CSBM feeding program had the least impact on climate change per 1,000 kg of carcass, followed by LP, compared with both DDGS feeding programs evaluated. In contrast, Shurson et al. (2022) reported that average greenhouse gas emissions (GHG; kg CO_2_ equiv.) per market hog produced were less (176.8 kg CO_2_ equiv.) when feeding low CP diets supplemented with CAA than when feeding CSBM diets (185.6 kg CO_2_ equiv.) to growing-finishing pigs regardless of geographic region in the United States. The primary reason for opposite results from these two studies is due to differences in LCA estimates used for corn (0.4258 vs. 0.571 kg CO_2_ equiv/kg, respectively) and soybean meal (0.48 vs. 1.062 kg CO_2_ equiv/kg, respectively). Another reason for these differences is that Shurson et al. (2022) used a highly specialized, spatially explicit Food System Supply-Chain Sustainability model to quantify GHG emissions, water consumption, and land use of corn, soybean meal, and DDGS based on county level sourcing, whereas state-level data were used in the Opteinics model, with corn and soybean data obtained from the GFLI database. Because environmental impacts of corn and soybean production vary across states and regions in the United States (Smith et al., 2017; Pelton, 2018; Brauman et al., 2020; Pelton et al., 2021), geographic region must be considered in LCA assessments of feed ingredients used in commercial swine feeding programs. Therefore, this is a limitation of environmental impact results provided by the Opteinics model. Inconsistent results have been reported in other studies that compared the impacts of feeding LP diets with CSBM diets on global warming potential. For example, Monteiro et al. (2017) observed a 4% decrease in global warming potential when feeding LP diets compared with conventional diets, while Esteves et al. (2021) reported no differences in global warming potential when reducing dietary CP from 18.15% to 15.15%. These inconsistent effects of feeding LP diets are due to differences in LCA attribution, system boundaries, types of ingredients used in diets, as well as differences in growth performance and manure composition.

Benavides et al. (2020) conducted an LCA study to compare environmental impacts of similar types of diets for swine and poultry and assumed carbon intensity values of 2,714 to 6,785 g CO_2_/kg ingredient for CAA and 558 to 869 g CO_2_/kg ingredient for DDGS, which had significantly greater carbon intensity than corn (304 g CO_2_/kg ingredient) and SBM (400 to 482 g CO_2_/kg ingredient). As a result, these authors reported a 20% increase in GHG emissions when feeding a 31.8% DDGS diet compared with feeding a standard CSBM diet, which is comparable to values reported in the current study.

Differences in acidification and eutrophication potential among feeding programs evaluated in the current study were relatively small. The CSBM feeding program had the least impact on marine eutrophication and freshwater eutrophication, while the LP feeding program had the least impact on acidification and terrestrial eutrophication among feeding programs. Feed production is the major contributor for eutrophication (>90%), and acidification (>40%) in pork production systems (Stone et al., 2012). Excretion of N, particularly the emission of NO_x_ and NH_3_, and the use of fossil fuel during feed production are associated with acidification, while excessive P excretion in manure can lead to eutrophication in various aquatic environments (Carpenter, 2008; Stone et al., 2012). Monteiro et al. (2017) reported that feeding LP diets resulted in 8% less acidification and 9% less eutrophication compared with feeding conventional CSBM diets. Similarly, Esteves et al. (2021) reported that reducing dietary CP from 18.15% to 15.15% and supplementing diets with CAA reduced acidification potential by 10% and reduced eutrophication potential by 14%. However, Mackenzie et al. (2016) reported less than a 2% difference on acidification and eutrophication potential when 26% DDGS was added to diets. These results show that the acidification and eutrophication potential of pork production systems is affected by diet composition and formulation method, manure management system, and the overall efficiency of dietary N and P utilization.

Impacts on water consumption for the production of various feed ingredients associated with feeding programs in the current study were the least per 1,000 kg carcass for the LP feeding program, while incorporating 30% DDGS into the diets substantially increased water use compared with LP and CSBM feeding programs. In contrast, Shurson et al. (2022) reported that the DDGS feeding program had less impact on water consumption per market hog (7.70 m^3^) than the CSBM feeding program (8.21 m^3^). The differences in results from similar diets evaluated in these two studies, are primarily due to differences in water consumption attributed to soybean and soybean meal production compared with DDGS production, which varied among pork production regions in the United States (Shurson et al., 2022). In addition, differences in allocation of the proportion of environmental impacts attributed to ethanol vs. DDGS and soybean oil vs. soybean meal affect relative difference is water consumption (Shurson et al., 2022). Corn and soybean production are water-intensive agricultural practices where water consumption of corn production can vary from 0.006 to 0.232 m^3^/kg and soybean meal production can vary from 0.014 to 0.210 m^3^/kg among geographic regions while DDGS production has less impact on water consumption which ranged from 0.007 to 0.160 m^3^/kg (Shurson et al., 2022). Benavides et al. (2020) also reported that use of DDGS in swine diets reduced water consumption compared with feeding CSBM diets.

The CSBM feeding program had slightly less impact on fossil resource use than the LP feeding program, both of which have less impact on fossil resource use than the two DDGS feeding programs in the current study. Similar results were also reported by Benavides et al. (2020) and Mackenzie et al. (2016) showing that including DDGS in swine diets significantly increased fossil resource use compared with feeding standard diets in the United States and western Canada, respectively. Because fossil resource use is one of the main causes of GHG emissions, similar trends of feeding program impacts were observed between the global warming potential and fossil resource use impacts. However, as previously discussed, the LCA values attributed to DDGS determines whether its use in CSBM diets is a detriment or a benefit. It is also important to recognize that although CAA have much greater carbon intensity than corn, soybean meal, and DDGS, their low dietary inclusion rates result in minimal effects on overall diet fossil resource use and other environmental impacts. Similar results were also reported by Benavides et al. (2020) and Mackenzie et al. (2016) showing that including DDGS in swine diets significantly increased fossil resource use.

All feeding programs that partially replaced SBM result in some benefit in reducing land use change because soybean yields per hectare are much less than for corn (Shurson et al., 2022) which results in soybean meal having a greater land use per kg of ingredient compared with corn and DDGS. Similar benefits of feeding diets containing relatively high amounts of CAA and DDGS on land use change have also been reported in other studies (Monteiro et al., 2017; Esteves et al., 2021). Thoma et al. (2016) reported that average land occupation to produce 1 kg of pig live BW in the United States was 4.22 m^2^a (square meters annum) but ranged from 4.11 to 4.59 m^2^a/kg live BW among geographic regions. These authors indicated that regional differences in land use in pork production were a result of differences in corn and soybean yields and climate, with pigs produced in regions with warmer climate having reduced feed intake, reduced growth rate, and extended feeding period to reach market weight. However, it is difficult to compare the results from Thoma et al. (2016) with results from the current study due to different systems boundaries and units of measure.

Using terminology for manure application as “residues” to provide nutrients to nutrient deficit agricultural land, and “waste” for manure application that exceeds soil needs for crop production, there were no differences in ranking of feeding programs among the various environmental impacts calculated. Relatively small differences were observed between manure “residue” and “waste” allocations for climate change, acidification, terrestrial eutrophication, marine eutrophication and particulate matter, but greater benefits were observed for using manure as a nutrient source (residue) for deficit soils. Although the purpose of this study was not to compare manure management practices, manure is the next most important factor affecting environmental impact of pork ­production systems because feeding programs directly affect N and P concentrations and emissions in manure (Shurson et al., 2022).

In summary, types of feed ingredients, relative differences in LCA environmental impacts, as well as diet formulation methods and composition affect productivity and efficiency of swine production systems which ultimately determine environmental impacts. The CSBM program evaluated in this study resulted in optimal growth performance, carcass composition, and reducing impacts on climate change, marine and freshwater eutrophication, and fossil resource use. However, the LP feeding program provided advantages of improving N utilization efficiency, and reducing acidification potential, terrestrial eutrophication, and water use, while the DDGS feeding programs had the least impact on land use.

These findings suggested that multiple perspectives, including productivity, efficiency, and environmental impacts, of feeding programs need to be considered when evaluating and developing more environmentally sustainable pork production systems. Special attention should be given to the assumptions used for LCA determinations of environmental impact measures of feed ingredients to ensure that they represent the most realistic and geographically specific crop, ingredient, and pork production region being considered due to substantial differences in environmental impacts.

## Supplementary Material

skad188_suppl_Supplementary_Table

## Abbreviations:

ADFI: average daily feed intake
ADG: average daily gain
BF: backfat
BUN: blood urea nitrogen
BW: body weight
CAA: crystalline amino acids
CDO: corn distillers oil
CP: crude protein
CSBM: corn-soybean meal diets
DDGS: distillers dried grains with solubles
FFL: fat-free lean
G:F: gain:feed
GHG: greenhouse gases
HCW: hot carcass weight
LCA: life cycle assessment
LMA: longissimus muscle area
LP: low protein CSBM diets supplemented with CAA
N: nitrogen
NE: net energy
P: phosphorus
SBM: soybean meal
SID: standardized ileal digestible
STTD: standardized total tract digestible
